# Pinocembrin alleviates memory impairment in transient global cerebral ischemic rats

**DOI:** 10.3892/etm.2014.1923

**Published:** 2014-08-19

**Authors:** FANRUI MENG, YUEHUA WANG, RUI LIU, MEI GAO, GUANHUA DU

**Affiliations:** 1 College of Pharmacy, Liaoning University, Shenyang, Liaoning 110036, P.R. China; 2 National Center for Pharmaceutical Screening, Institute of Materia Medica, Chinese Academy of Medical Sciences and Peking Union Medical College, Beijing 100050, P.R. China

**Keywords:** pinocembrin, transient global cerebral ischemia, cognition impairment, neuronal loss

## Abstract

The aim of the present study was to investigate the effect of pinocembrin on cognitive ability impairment in a rat model of transient global cerebral ischemia (TGCI). The TGCI model was established by inducing global cerebral ischemia for 20 min, followed by reperfusion for two weeks. The rats were divided into five experimental groups, including the sham group that were not subjected to ischemia, and four ischemic groups where the rats were exposed to TGCI. The sham and control TGCI groups were administered a vehicle intravenously immediately after reperfusion, while the other three groups were intravenously treated with 1, 5 and 10 mg/kg pinocembrin, respectively. In the present study, neurological scores were analyzed at 0 and 24 h after reperfusion, and the effect of pinocembrin on cognitive ability impairment in the TGCI rat model was investigated using a Morris water maze test. Neuronal loss was observed under an optical microscope with the assistance of Nissl staining. In addition, glial fibrillary acidic protein (GFAP)-positive cells were observed under an optical microscope by an immunohistochemistry assay. Pinocembrin treatment was found to alleviate the cognitive impairments, decrease the neurological scores, diminish neuronal loss in the hippocampus and reduce the number of GFAP-positive cells in the hippocampal CA1 region of the TGCI rats. Therefore, pinocembrin alleviated memory impairment in the TGCI rats.

## Introduction

Transient global cerebral ischemia (TGCI) leads to chronic memory impairment in clinical and animal models (1,2). TGCI in rats is a common animal model used for the study of cerebral ischemia-reperfusion injury (3). In the animal model, TGCI leads to severe neuronal damage in selectively vulnerable brain areas, such as the hippocampal CA1 region (4). With the aim to find an effective treatment for cerebral ischemia, research has been conducted using animal models to identify drugs that reduce the extent of the brain injury and cognitive impairment induced by cerebral ischemia-reperfusion injury (5). However, to date, no drug has been demonstrated to be effective in clinical trials (5).

Pinocembrin (5,7-dihydroxyflavanone) is a flavonoid compound that is abundant in propolis. Pinocembrin exhibits a variety of biological effects, including antitumor, antimicrobial, anti-inflammatory and vasorelaxative activity (6), and is easily transported through the blood-brain barrier (7). Previous studies revealed that pinocembrin protected against cerebral ischemic injury in rat models of middle cerebral artery occlusion and TGCI (8,9). Pinocembrin has also been shown to alleviate the damage of primary cultured cortical neurons and cerebral microvessel endothelial cells in oxygen-glucose deprivation/reoxygenation (10). Furthermore, the flavonoid has been demonstrated to protect the structure and function of cerebral mitochondria in a rat model of chronic cerebral hypoperfusion (11).

However, to the best of our knowledge, there are no behavioral studies on the therapeutic potential of pinocembrin in a TGCI model, where the rats have been subjected to global cerebral ischemia for 20 min, followed by reperfusion for two weeks. In the present study, the hypothesis that pinocembrin protects against cognitive impairment induced by TGCI was analyzed. The effects of pinocembrin on neurological scores, neuronal loss and the glial fibrillary acidic protein (GFAP)-positive cell count in the hippocampus were investigated with the aim of identifying the underlying mechanisms.

## Materials and methods

### Animals, surgical procedures and neurological scores

Adult male Sprague-Dawley rats (body weight, 300±30 g) were obtained from the Vital River Company (Beijing, China). Prior to surgery, the rats fasted for 4 h and had free access to water. All the procedures obeyed the Chinese Academy of Medical Sciences and Peking Union Medical College (Beijing, China) guidelines and ethics for animal experiments.

The rats were divided into five experimental groups (n=50). The sham group (n=10) consisted of rats that had not been exposed to ischemia, whereas the other four groups (n=10 per group) comprised rats that had been exposed to TGCI. The sham and control TGCI groups were administered a vehicle (hydroxypropyl-β-cyclodextrin) intravenously immediately after reperfusion. The other three groups were intravenously treated with 1, 5 and 10 mg/kg pinocembrin, respectively. Pinocembrin was synthesized and processed as sterile injection powder at the Department of New Drug Development, Institute of Materia Medica, Chinese Academy of Medical Sciences. The rats continued to be administered pinocembrin or the vehicle intravenously once a day at the same time until they were sacrificed (9).

The rats were anesthetized with 10% chloral hydrate (4 ml/kg) and the surgical procedures were performed as described in previous studies (4,9). The two common carotid arteries were isolated from the surrounding tissue, and the vertebral arteries were occluded by electric coagulation. After recovery for 24 h, the rats were anesthetized with ether, and the common carotid arteries were occluded for 20 min and reperfused. The body temperature of the rats was maintained at 37.0±0.5°C. Rats in the sham group were treated similarly to the ischemic groups, but without blocking the arteries. Immediately after reperfusion, the rats were given neurological scores on a scale up to 25 (4,9). Rats with a neurological score of ≥10 were considered successful; thus, participated in the following experiments. The rats were scored again at 24 h following reperfusion.

### Behavioral assessment

After reperfusion for 14 days, the spatial learning and memory of the rats were tested in an open-field Morris water maze (diameter, 150 cm; height, 50 cm). The pool was filled with water at a temperature of 25±1°C, and a circular platform (diameter, 10 cm; position, 1.5 cm below the water surface) was placed in the first quadrant. During a training trial, each rat had to escape the water by climbing onto the circular platform, which was not visible to the rat. The time the rat took to locate the platform was recorded. If the rat failed to locate the platform within 90 sec, the trial was terminated and the unsuccessful rat was guided onto the platform. All the rats were allowed to remain on the platform for 20 sec. After a final training trial on day 5, the rats were subjected to a probe trial (60 sec) in the maze without the presence of a platform. The length of time that the rats remained in the first quadrant was recorded. All the trials were recorded for analysis with a computer-assisted image analyzer (11,12).

### Nissl staining

Following the behavioral test, the rats were anesthetized, perfused with 0.9% NaCl (1,000 ml/kg) and reperfused with 4% paraformaldehyde (1,000 ml/kg; n=4 per group). The brains were removed and fixed in the same solvent (4% paraformaldehyde), and embedded in paraffin. Coronal sections with a thickness of 5 μm were cut for Nissl staining, as described previously (4). The samples were photographed under a light microscope (Olympus Corp., Tokyo, Japan) (magnification, ×40 and ×400). The neuronal density was determined by the average number of surviving hippocampal CA1 and CA4 neurons per 1-mm section, with three sections of bilateral hippocampal slices (4,7).

### Immunohistochemistry assay

Brain samples embedded in paraffin were cut into 5-μm thick coronal sections for the immunohistochemistry assay. Free floating sections were incubated at 4°C for 24 h in a mixture of 0.05 M phosphate-buffered saline (PBS), containing mouse anti-GFAP (1:1,000 dilution; Santa Cruz Bioctechnology, Inc., Santa Cruz, CA, USA), 0.3% Triton X-100 and 1% bovine serum albumin, as described previously (13). The sections were subsequently incubated for 90 min with a secondary antibody labeled with biotin (1:200), following which the sections were treated with 0.02% 3,3′-diaminobenzidine and 0.01% H_2_O_2_ for 3 min. Next, the sections were washed three times with PBS for 5 min. Finally, the sections were mounted on gelatin-coated slides, dehydrated in an ascending alcohol series, cleared in xylene (13) and photographed under a light microscope (magnification, ×400).

### Statistical analysis

All data are presented as the mean ± standard error of mean. The behavioral studies performed in the Morris water maze were evaluated with two-way analysis of variance (ANOVA) and the Bonferroni post hoc test. Other data were evaluated with one-way ANOVA and the Newman-Keuls post hoc test. P<0.05 was considered to indicate a statistically significant difference.

## Results

### Pinocembrin decreases the neurological scores of TGCI rats

**Figure 1:**
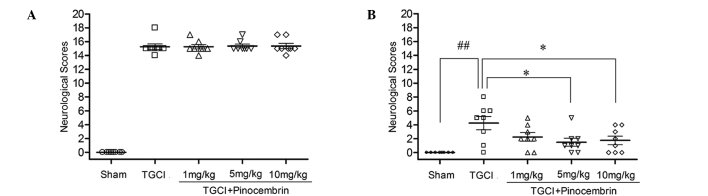
Pinocembrin decreased the neurological scores in TGCI rats. Neurological scores at (A) 0 h and (B) 24 h after reperfusion. Data are expressed as the mean ± standard error of mean. ^##^P<0.001, vs. sham group; ^*^P<0.05, vs. TGCI group (n=8). TGCI, transient global cerebral ischemia.

As shown in Fig. 1, the neurological scores of the sham rats were zero, indicating no neurological deficits. However, the scores of the other four groups increased following global cerebral ischemia-reperfusion compared with those of the sham group (P<0.001), and there were no statistically significant differences among the ischemic group scores (Fig. 1A). The scores of the TGCI group at 24 h after reperfusion increased compared with the sham group (P<0.001), while the scores of the pinocembrin 5 and 10 mg/kg groups decreased when compared with the control group (P<0.05; Fig. 1B). Since the neurological score indicates the extent of brain damage, the data indicated that pinocembrin reduced the neurological symptoms in the TGCI rats.

### Pinocembrin reduces cognitive impairment in TGCI rats

**Figure 2:**
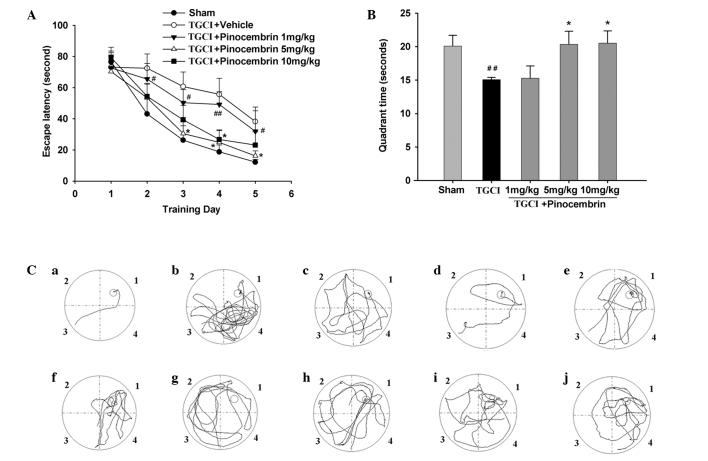
Effect of pinocembrin on spatial learning and memory in TGCI rats using a Morris water maze test. (A) Mean daily latency of escaping from the first trial with the hidden platform to the fifth trial day. (B) Time spent in quadrant 1 during the 60 sec probe trial (no platform). (C) Representative swimming paths on the (a–e) fifth training day and (f–j) probe trial in the (a and f) sham, (b and g) TGCI, (c and h) 1 mg/kg pinocembrin, (d and i) 5 mg/kg pinocembrin and (e and j) 10 mg/kg pinocembrin groups. Data are expressed as the mean ± standard error of mean. ^#^P<0.05 and ^##^P<0.01, vs. sham group; ^*^P<0.05, vs. TGCI group (n=8). TGCI, transient global cerebral ischemia.

Behavioral deficits are the major sequela in patients that have suffered a stroke, particularly impairment in learning and memory after cerebral ischemia. In order to investigate the effects of pinocembrin on the memory damage caused by the TGCI procedure, the learning and memory of the rats were assessed in the Morris water maze 14 days after TGCI. During the five-day training period of the hidden platform test, the mean latency to locate the platform (escape latency) decreased progressively in all the groups. Rats in the TGCI group required additional time to locate the platform when compared with the rats in the sham group and those that had been administered 5 and 10 mg/kg pinocembrin (Fig. 2A). In the probe test, the time the control group rats stayed in quadrant 1 (where the platform was located) decreased compared with the sham group rats, while the time that the pinocembrin-treated rats spent in quadrant 1 increased (Fig. 2B). Fig. 2C demonstrates the representative swimming paths of all the rats in a group, indicating their training performance on day 5 of the trials in the presence of the platform (a–e) and their probe trial performance in the absence of the platform (f–j). The results revealed that the memory injury of the rats treated with pinocembrin was alleviated compared with the TGCI group rats.

### Pinocembrin reduces neuronal loss in TGCI rats

**Figure 3:**
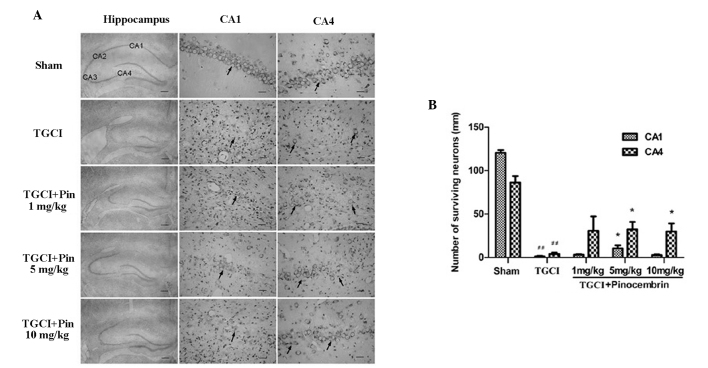
Pinocembrin attenuated neuronal loss in the hippocampal CA1 and CA4 regions in TGCI rats. (A) Representative images of Nissl-stained CA1 and CA4 areas of the hippocampus (hippocampus magnification, ×40; CA1 and CA4 areas magnification, ×400). (B) Neuronal density of the hippocampal CA1 and CA4 regions. Data are expressed as the mean ± standard error of mean. ^##^P<0.01, vs. sham group; ^*^P<0.05, vs. TGCI group (n=4). TGCI, transient global cerebral ischemia.

Neurons are easily damaged during cerebral ischemia, particularly in the hippocampal CA1 region. In this study, the neurons of the sham group showed clear nuclei and nucleoli, while the TGCI rats exhibited significant neuronal damage in the hippocampal CA1 and CA4 regions when compared with the sham group. In addition, neurons in the ischemic areas showed significantly pyknotic nuclei (Fig. 3A). Neurons were also counted in the hippocampal CA1 and CA4 regions, and TGCI was shown to destroy ~99% of the neurons in the CA1 region and 75% of the neurons in the CA4 region in the control group rats. However, pinocembrin reduced neuronal death in the hippocampal CA1 and CA4 regions in the TGCI rats (Fig. 3B).

### Pinocembrin reduces the amount of GFAP-positive cells

**Figure 4:**
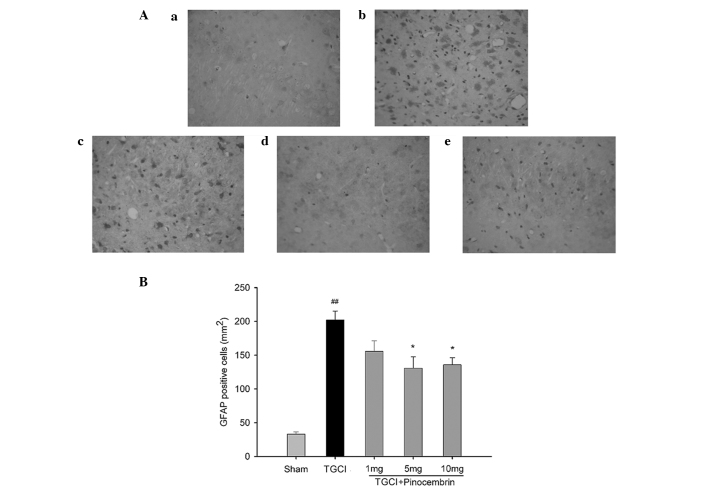
Pinocembrin attenuated astrocyte proliferation in TGCI rats. (A) Representative images of the hippocampal CA1 region (magnification, ×400) in the (a) sham, (b) control, (c) 1 mg/kg pinocembrin, (d) 5 mg/kg pinocembrin and (e) 10 mg/kg pinocembrin groups. (B) Pinocembrin reduced the astrocyte cell count in the hippocampal CA1 region of TGCI rats. Values are expressed as the mean ± standard error of mean. ^##^P<0.01, vs. sham group; ^*^P<0.05, vs. TGCI group (n=4). TGCI, transient global cerebral ischemia.

Astrocyte cells are activated and an inflammatory response is aggravated following cerebral ischemia-reperfusion injury. The proliferation of astrocytes, as indicated by the count of GFAP-positive cells, increased in the TGCI rats, while the effects were decreased in the TGCI rats that were administered 5 and 10 mg/kg pinocembrin. Fig. 4A shows representative images of the GFAP immunohistochemistry assay, and Fig. 4B shows the GFAP-positive cell count.

## Discussion

Previous studies have demonstrated that pinocembrin protects neurons against cerebral ischemia-reperfusion injury and attenuates the disruption of the blood-brain barrier in TGCI rats (13,14). In the present study, pinocembrin was demonstrated to decrease memory impairment, neurological scores, neuronal loss in the hippocampus and the number of GFAP-positive cells in the hippocampal CA1 region of TGCI rats.

In an ischemic rat model, the rats develop TGCI features due to the occlusion of the bilateral vertebral and common carotid arteries, and ischemic damage in the hippocampus results in memory impairment (15). A Morris water maze test is often performed to evaluate the damage in the hippocampus and spatial memory impairment (13,16). Ischemic damage in the TGCI rats led to an impairment of spatial learning and memory, as revealed by behavioral tests. The observations of learning and memory impairment and the morphological changes following TGCI injury were consistent with previous studies (4,16,17). Behavioral tests demonstrated that the TGCI rats treated with pinocembrin performed better in the water maze when compared with the untreated TGCI rats. It was also evident that pinocembrin reduced the extent of ischemic neuronal damage in the hippocampal CA1 region. Thus, the data demonstrated that pinocembrin treatment resulted in marked behavioral protection against TGCI injury.

Neuronal death following global ischemia occurs in a delayed manner (18), which is commonly referred to as delayed neuronal death. In the present study, the extent of neuronal death in the hippocampal CA1 region in the control and pinocembrin-treated groups was increased compared with the neuronal death observed in a previous study following reperfusion for 24 h (4). The production of reactive oxygen species (ROS) is enhanced, and the subsequent oxidative stress and inflammatory reactions may play an important pathological role in ischemia-reperfusion (3,4). In TGCI, the increased formation of ROS has been regarded as an underlying factor for mediating delayed neuronal death, particularly for neurons in the hippocampal CA1 region (19). An increased level of ROS damages the cell membranes and other cellular structures, disturbs mitochondrial function and leads to the release of mitochondrial cytochrome *c* and the activation of apoptotic pathways (20,21). Pinocembrin alleviates oxidative stress; therefore, reduces TGCI-induced injury (4). The present study demonstrated that pinocembrin alleviated neuronal loss in the hippocampal CA1 region in the TGCI rats.

Neuroprotection may be assessed by the neuron count in the hippocampal CA1 region, and is essential for the evaluation of neuroprotective effects using behavioral and histological measures (22,23). The present study confirmed that the application of global cerebral ischemia for 20 min in rats resulted in damage to >99% of the neurons in the hippocampal CA1 region. As revealed histologically, a significant impairment in learning and memory in the TGCI rats was indicated by a poor performance in the Morris water maze.

It has been established that inflammatory reactions are responsible for neuronal damage in cerebral ischemia, including focal and global ischemia (7,24). A number of investigations have demonstrated the critical role of inflammation after ischemia and reperfusion in stroke (7,8). The inflammatory events occur at the blood-endothelium interface of the cerebral capillaries, and aggravate the ischemic tissue damage in order to improve the blood-brain barrier permeability (5,9). The activation of microglia and astrocyte cells aggravates the injury caused by cerebral ischemia. In the present study, astrocyte cells were shown to proliferate in the TGCI rats; however, the proliferation effect was reduced in the rats that were administered 5 and 10 mg/kg pinocembrin. Therefore, the effects of pinocembrin on astrocyte cells of TGCI rats may reduce neuronal damage.

In conclusion, the present study revealed that pinocembrin alleviated memory impairment in TGCI rats. This may be attributed to the effect of pinocembrin against neuronal damage and astrocyte proliferation in TGCI rats.

## Abbreviations

TGCI: transient global cerebral ischemia
GFAP: glial fibrillary acidic protein
ROS: reactive oxygen species
PBS: phosphate-buffered saline
ANOVA: analysis of variance
